# Morphometric Study of the Human Cadaveric Spleen: Dimensions, Shape Variations, and the Prevalence of Border Notches and Fissures

**DOI:** 10.7759/cureus.112655

**Published:** 2026-07-14

**Authors:** Aditya A Musale, Kinjal Jethva, Hetal Vaishnani, Bhoopesh Raja

**Affiliations:** 1 Department of Anatomy, Smt. B. K. Shah Medical Institute and Research Centre, Sumandeep Vidyapeeth Deemed to Be University, Vadodara, IND

**Keywords:** anatomical variation, cadaveric study, spleen, splenectomy, splenic fissure, splenic morphometry, splenic notch

## Abstract

Introduction: The spleen is a secondary lymphoid organ. Splenic notches and fissures are remnants of incomplete fusion of embryonic lobules. The spleen is considered a vital organ in the immune system. It plays a crucial role in filtering blood, removing old or damaged red blood cells, and producing antibodies to fight off infections. The spleen also acts as a reservoir for blood, storing platelets and white blood cells for release in times of need. Due to its important functions, the spleen exhibits considerable variation in size, shape, and surface features among individuals.

Materials and methods: This prospective, cross-sectional, observational study examined 20 formalin-fixed adult cadaveric spleens in a tertiary teaching institute. Weight was recorded on a digital balance, while length (maximal pole-to-pole distance) and breadth (maximal superior-to-inferior border distance) were measured with extended-jaw Vernier calipers. Each measurement was taken three times and averaged to minimize observer bias. Shape was classified as wedge, tetrahedral, or irregular; notches and fissures were recorded separately on the superior and inferior borders, with fissures graded as incomplete or complete. Proportions are reported as frequencies with 95% confidence intervals (CIs), and superior- versus inferior-border prevalences were compared using McNemar’s test.

Results: The mean weight was 91.35 ± 42.26 g (range 28-197 g), mean length 87.43 ± 19.49 mm (range 51.5-118.4 mm), and mean breadth 58.04 ± 10.74 mm (range 35.8-79.9 mm). The wedge shape predominated (11/20; 55%), followed by tetrahedral (8/20; 40%) and irregular (1/20; 5%). Notches were frequent on both borders, occurring on the superior border in 15 specimens (75%) and the inferior border in 14 (70%). Fissures were more common on the superior border (8/20; 40%, all incomplete) than the inferior border (4/20; 20%); a single complete fissure (1/20; 5%) was observed on the inferior border.

Conclusions: Cadaveric spleens demonstrate notable fissural variation; the morphometry of the spleen is a crucial aspect of understanding its structure and function. Through our research, we have gained valuable insights into the size, shape, and distribution of various components within the spleen. Familiarity with these normal variants helps radiologists and surgeons avoid misinterpreting them as splenic injury or disease and is a useful adjunct to anatomical teaching.

## Introduction

The spleen is a large, encapsulated mass of vascular and lymphoid tissue situated in the left hypochondrium between the fundus of the stomach and the diaphragm. As the largest secondary lymphoid organ, it contributes to immune surveillance, filtration of senescent erythrocytes, and storage of blood cells. Its shape is classically described as varying from a curved wedge to a domed tetrahedron, with textbook dimensions of approximately 120 mm in length, 70 mm in breadth, and 40 mm in thickness, and a weight commonly cited between 80 and 300 g. These values are influenced by age, sex, nutritional status, and individual variation, so textbook ranges may not reflect a given population [1,2]. Contemporary imaging series confirm that splenic size is significantly influenced by body height and sex and correlates with anthropometric body parameters [3,4].

Embryologically, the spleen arises during the fifth week of intrauterine life from several mesenchymal condensations in the dorsal mesogastrium. These lobules normally fuse into a single organ before birth; when fusion is incomplete, the separations persist as notches along the borders or as deeper clefts termed fissures. Such notches and fissures are therefore developmental remnants rather than acquired lesions, but on cross-sectional imaging or at operation, they can closely mimic splenic lacerations, infarcts, or accessory lobes [5,6]. The classical account of lobar fusion has more recently been re-examined: three-dimensional reconstruction of the early fetal spleen demonstrates a single primordium [7], and a radiological study found no evidence for a multifocal or lobulated developmental origin, suggesting that surface notches may instead reflect folding of the capsule rather than persistent inter-lobar clefts [8]. Incomplete separation of splenic tissue may likewise give rise to accessory spleens, whose embryological basis and prevalence have been revisited [9].

A clear understanding of normal splenic morphology and its variations is consequently relevant to several disciplines: it assists surgeons in planning splenectomy and trauma repair, helps radiologists distinguish physiological variants from pathology, and underpins anatomical education [10,11]. Against this background, the present study was undertaken to document the morphometry and shape of adult cadaveric spleens and to determine the prevalence and border-wise distribution of splenic notches and fissures in a regional cadaveric sample. These considerations are reinforced by surgical and radiological series in which accessory spleens and variant splenic artery and hilar anatomy must be anticipated during splenectomy or spleen-preserving procedures [12,13].

## Materials and methods

Study design and setting

This was a prospective, cross-sectional, descriptive, observational study conducted in the Department of Anatomy, Smt. B.K. Shah Medical Institute and Research Centre, Sumandeep Vidyapeeth (Deemed to be University), Vadodara, India. The study concept, literature review, and refinement of the materials and methods were completed before ethical approval. Following approval from the Sumandeep Vidyapeeth Institutional Ethics Committee (SVIEC) on 18/06/2026, specimen data were collected between 19/06/2026 and 24/06/2026.

Ethical considerations

The study was approved by the SVIEC (outward ref.: SVIEC/0N/medi/SRP/June/26/212; dated June 18, 2026). All cadaveric material was obtained and handled in accordance with institutional ethical guidelines, with due respect for donor dignity and confidentiality. The spleens studied were obtained from embalmed teaching cadavers available in the Department of Anatomy and were used for research in accordance with the institutional policies governing the use of human cadaveric material.

Specimens and sampling

Twenty (n = 20) formalin-fixed adult human cadaveric spleens obtained from previously dissected embalmed cadavers used for routine undergraduate teaching and from the Department of Anatomy organ-storage repository were studied. Purposive sampling was used, the sample size being determined by specimen availability.

The sample size was determined by the availability of suitable cadaveric specimens (convenience sampling); no a priori power calculation was performed, as is customary in descriptive cadaveric morphometric studies. For context, the single-proportion formula $n = \frac{\mathrm{Z}^2\mathrm{P}(1-\mathrm{P})}{\mathrm{d}^2}$ (where Z = 1.96 corresponds to 95% confidence, P is the expected proportion of a given feature, and d is the absolute precision) [14] indicates that estimating a feature prevalence of 50%-75% with an absolute precision of ±20% would require approximately 19-25 specimens. The achieved sample of 20 spleens, therefore, lies within this range and is of an order comparable to previously published cadaveric spleen morphometry series [1,10]. To convey the precision attainable with this sample, all prevalence estimates are reported with 95% confidence intervals (CIs).

Inclusion criteria

Adult human cadaveric spleens with an intact capsule and clearly identifiable borders, poles, notches, and fissures, fixed in 10% formalin, were included in the study.

Exclusion criteria

Cadavers with spleens that were damaged, ruptured, traumatised, or distorted at the poles, or that had undergone surgical alteration or dissection artefact compromising clear visualisation were excluded.

Measurements and parameters

Each spleen was examined for the following parameters: Weight (g): recorded on a digital weighing balance; Length (mm): the maximum distance between the two poles along the longitudinal axis, measured with extended-jaw Vernier calipers; Breadth (mm): the maximum distance between the superior and inferior borders, measured perpendicular to the length; Shape: classified by visual inspection as wedge, tetrahedral, or irregular; Notches: counted separately on the superior and inferior borders; Fissures: identified separately on the superior and inferior borders and graded as incomplete (a partial cleft) or complete (a cleft dividing the organ).

To reduce observer bias, every linear measurement was recorded three times, and the mean value was used for analysis. The materials used comprised 10% formalin-fixed spleen specimens, extended-jaw Vernier calipers, a digital weighing balance, and a high-resolution camera for documentation.

Statistical analysis

Data were entered into a Microsoft Excel spreadsheet (Microsoft Corp., Redmond, WA, USA) and analysed using descriptive and inferential statistics. Continuous variables (weight, length, breadth) are expressed as mean ± standard deviation (SD) with ranges, and categorical variables (shape, notches, fissures) as frequencies and percentages; 95% CIs for categorical proportions were calculated using the Wilson score method. Because both borders were assessed on the same specimens, the prevalence of notches and of fissures on the superior versus the inferior border was compared using McNemar’s test for paired nominal data. A two-tailed p-value < 0.05 was considered statistically significant. Statistical analyses were performed using IBM SPSS Statistics, version 26.0 (IBM Corp., Armonk, NY, USA).

## Results

**Figure 1:**
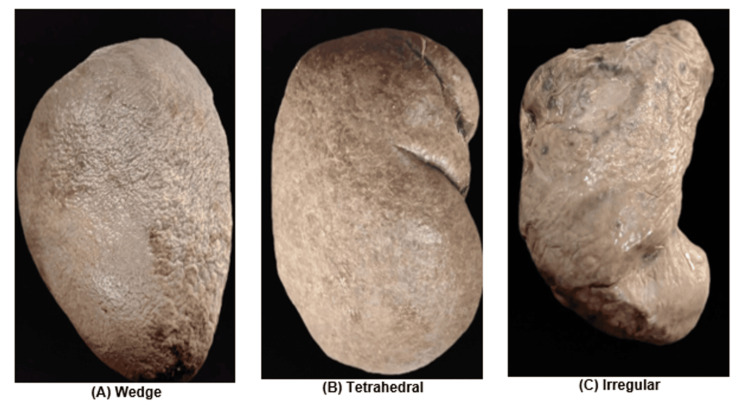
Representative morphological shapes of the cadaveric spleen. (A) Wedge shape; (B) Tetrahedral shape; (C) Irregular shape Photographs of the specimens obtained by the authors.

**Figure 2:**
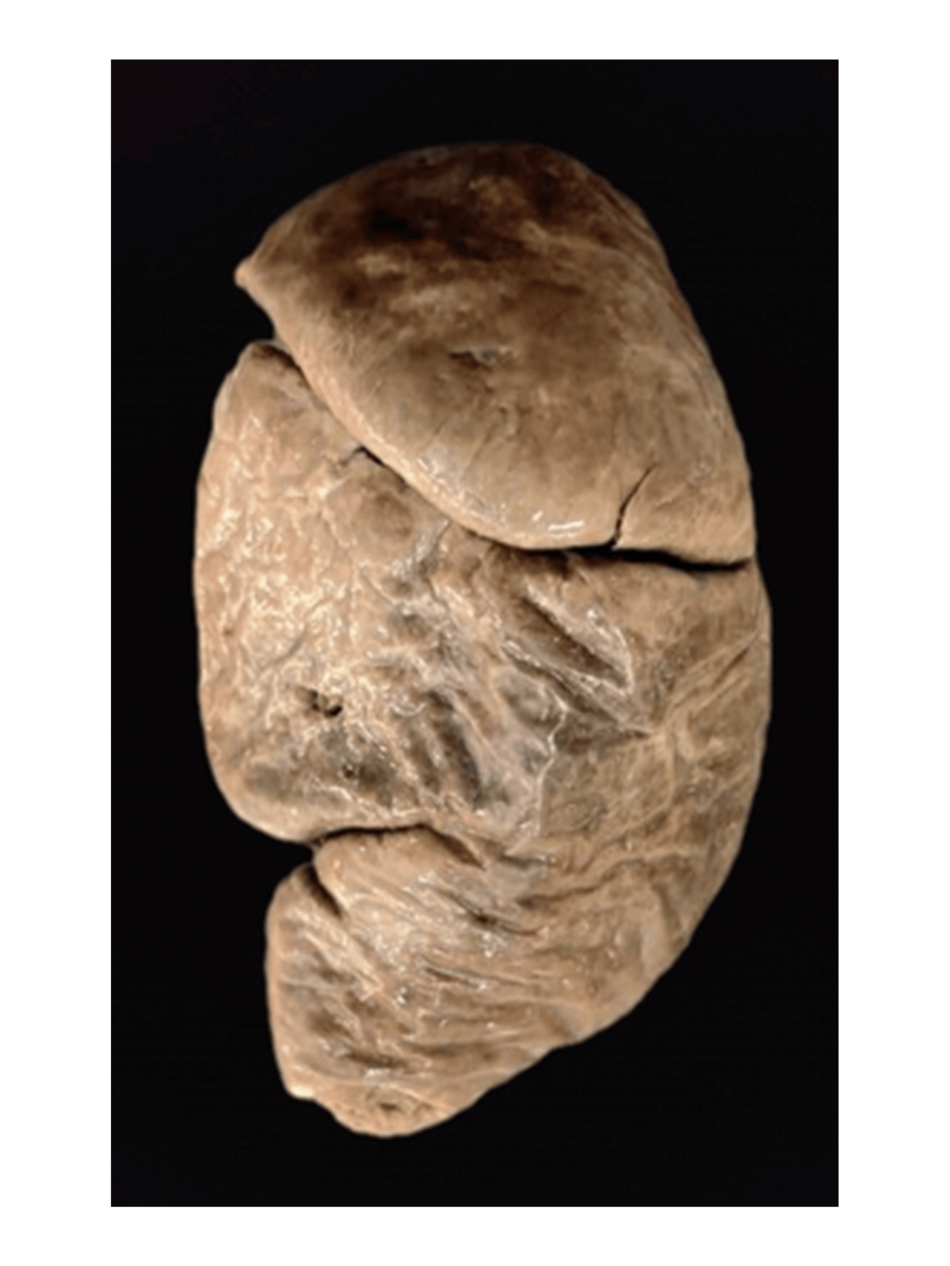
Cadaveric spleen demonstrating a complete fissure dividing the organ. Photograph of the specimen obtained by the authors.

A total of 20 cadaveric spleens were analysed; the specimen-wise data are presented in Table 1. Representative morphological forms and an example of a complete fissure are shown in Figures 1-2.

**Table 1:** Specimen-wise morphometric and surface-feature data of 20 cadaveric spleens.

Serial number	Shape	Weight (g)	Length (mm)	Breadth (mm)	Superior notch (n)	Inferior notch (n)	Superior fissure	Inferior fissure
1	Wedge	160	115.4	74.6	4	1	Two incomplete	None
2	Tetrahedral	135	110.66	69.2	4	1	Two incomplete	None
3	Wedge	97	85.1	63.5	0	2	None	None
4	Wedge	197	114.6	79.9	1	0	None	None
5	Tetrahedral	101	88.6	63.8	0	2	None	Two incomplete
6	Tetrahedral	66	83.7	54.2	2	0	Two incomplete	None
7	Tetrahedral	81	91.7	56.6	1	1	One incomplete	One complete
8	Tetrahedral	70	72.8	52.7	2	1	None	None
9	Wedge	73	77.4	63.2	2	2	None	Two incomplete
10	Wedge	65	77.5	53.2	1	0	One incomplete	None
11	Tetrahedral	28	51.5	44.6	1	0	One incomplete	None
12	Wedge	55	66.3	46.9	1	2	None	None
13	Irregular	36	58.7	35.8	2	0	None	None
14	Tetrahedral	133	96.2	61.7	1	1	None	None
15	Wedge	82	68.3	55.7	0	1	None	None
16	Wedge	78	118.4	55.5	1	1	One incomplete	None
17	Wedge	48	73.6	46.7	2	0	Two incomplete	None
18	Tetrahedral	85	103.5	50.2	1	2	None	One incomplete
19	Wedge	117	90.4	67.6	0	2	None	None
20	Wedge	120	104.3	65.1	0	1	None	None

The mean weight was 91.35 ± 42.26 g (range 28-197 g). The mean length was 87.43 ± 19.49 mm (range 51.5-118.4 mm) and the mean breadth 58.04 ± 10.74 mm (range 35.8-79.9 mm) (Table 2). These values show wide variability, with the heaviest spleen (197 g) weighing almost seven times as much as the lightest (28 g).

**Table 2:** Summary of splenic morphometric parameters (n = 20).

Parameter	Mean ± SD	Range
Weight (g)	91.35 ± 42.26	28–197
Length (mm)	87.43 ± 19.49	51.5–118.4
Breadth (mm)	58.04 ± 10.74	35.8–79.9

The wedge shape was the most common, seen in 11 spleens (55%; 95% CI 34.2-74.2), followed by the tetrahedral shape in eight (40%; 95% CI 21.9-61.3); a single specimen (5%; 95% CI 0.9-23.6) was irregular (Table 3).

**Table 3:** Distribution of splenic shapes (n = 20).

Shape	Number of specimens (n)	Prevalence (%)
Wedge	11	55
Tetrahedral	8	40
Irregular	1	5
Total	20	100

Notches were common on both borders. A superior border notch was present in 15 spleens (75%; 95% CI 53.1-88.8) and an inferior border notch in 14 (70%; 95% CI 48.1-85.5), with total notch counts of 26 on the superior and 20 on the inferior border. The difference in notch prevalence between the two borders was not statistically significant (McNemar’s test, p = 1.00). Fissures were more frequent on the superior border, where they occurred in eight specimens (40%; 95% CI 21.9-61.3) and were exclusively incomplete. Inferior border fissures were present in four specimens (20%; 95% CI 8.1-41.6), of which three were incomplete and one was complete. The higher prevalence of superior-border fissures did not reach statistical significance (McNemar’s test, p = 0.34). The single complete fissure (specimen 7; 5% of the sample) divided the inferior aspect of the organ and was the only complete cleft in the series (Table 4).

**Table 4:** Border-wise prevalence of notches and fissures (superior border in the left column, inferior border in the right column).

Feature	Superior border, n (%)	Inferior border, n (%)
Specimens with notches	15 (75.0)	14 (70.0)
Total notches counted	26	20
Specimens with fissures	8 (40.0)	4 (20.0)
Incomplete fissures	8 (40.0)	3 (15.0)
Complete fissures	0 (0.0)	1 (5.0)

Because the specimens were examined in isolation and not in situ, positional variants such as wandering (ectopic) spleen and accessory spleens (splenunculi) could not be systematically assessed; no such findings were documented among the specimens included in this study.

## Discussion

This cadaveric study of 20 adult spleens documented wide morphometric variability, a predominance of the wedge shape, and a high frequency of border notches and incomplete fissures. These observations are broadly concordant with the existing literature, while differing in some respects that merit comment.

The mean weight in the present series (91.35 g) was lower than several previously reported cadaveric means, such as 145 g reported by Setty and Katikireddi and 149.33 g by Sangeeta et al. [2,5]. Lower cadaveric weights are commonly attributed to prolonged formalin fixation, drainage of splenic blood, and population and nutritional differences; the wide weight range observed here (28-197 g) is consistent with the organ’s well-recognized plasticity. The mean length (87.43 mm) and breadth (58.04 mm) were likewise smaller than the often-quoted textbook figures of 120 mm and 70 mm, again in keeping with fixation-related shrinkage and sampling variation. The wide weight range observed here (28-197 g) is also consistent with the broad reference intervals documented in large contemporary organ-weight databases [15].

The predominance of the wedge shape (55%) accords with the findings of Chaware et al., Gujar et al. (52%) and Baimai et al. (46.75%), all of whom reported the wedge form as the commonest morphology [1,10,16]. The tetrahedral form was the second most frequent in the present study (40%), broadly comparable with the proportions described in those series.

Superior border notches were frequent (75%), in close agreement with reported prevalences of 78% (Chaware et al.), 86.6% (Sangeeta et al.), 72% (Gujar et al.), and up to 70% (Baimai et al.) [1,5,10,16]. A notable feature of the present series was the high prevalence of inferior border notches (70%), which is considerably greater than the 10%-15% typically reported. This may reflect the counting of all visible notches (including shallow ones) on both borders, rather than only prominent superior notches, as well as genuine population variation; it underscores that the inferior border is not exempt from notching and should be inspected with equal care.

Fissures followed the expected pattern, being commoner on the superior border and predominantly incomplete, with complete fissures rare. Only one complete fissure (5%) was identified on the inferior border. This is consistent with Arumugam and Subbiah, who described incomplete fissures more frequently than complete ones and noted that complete clefts producing a bilobed spleen are rare congenital variants [6]. Recognition of these clefts is clinically important because a deep fissure can be misread as a laceration on contrast-enhanced computed tomography in the trauma setting.

The clinical relevance of these findings is threefold. For surgeons, awareness of border notches, fissures, and shape variants reduces the risk of misidentifying normal anatomy as pathology and helps anticipate variant hilar anatomy during splenectomy or partial splenic resection. For radiologists, normative cadaveric data assist in distinguishing physiological notches and fissures from lacerations, infarcts, and accessory spleens, thereby reducing false-positive interpretations [11]. Finally, the developmental basis of notches and fissures as fusion lines reinforces their value as teaching material in anatomy and embryology. Standardized ultrasound morphometry protocols [17] and population-specific normative datasets, such as those reported for the North Indian population [18], can further support this distinction in routine practice.

Limitations

The principal limitations are the small, purposively sampled cadaveric series (n = 20) and the fact that the specimens were obtained from previously dissected teaching cadavers and the departmental organ-storage repository, so complete demographic information (age and sex) was not available or could not be reliably linked to individual specimens; this precluded analysis of demographic associations. Formalin fixation may have altered absolute weight and dimensions, so the values reported here should be applied with caution to living populations. As the spleens were examined in isolation rather than in situ, positional variants (wandering spleen) and accessory spleens (splenunculi) could not be systematically assessed. Analyses were largely descriptive, with only limited paired comparisons between the two borders undertaken. Larger studies recording demographic variables and correlating cadaveric with radiological measurements would strengthen the normative value of these data.

## Conclusions

In this cadaveric series, adult human spleens showed marked variability in weight and dimensions, a predominance of the wedge shape, and a high prevalence of notches on both borders together with predominantly incomplete fissures of the superior border. Complete fissures were rare. Because these normal anatomical variants can closely simulate traumatic or pathological lesions, familiarity with their prevalence and border distribution is valuable for accurate radiological interpretation, safe splenic surgery, and effective anatomical teaching.
